# An acid trip activates protumoral macrophages to promote hepatocellular carcinoma malignancy

**DOI:** 10.1172/JCI158562

**Published:** 2022-04-01

**Authors:** Nicola Graham, Jeffrey W. Pollard

**Affiliations:** MRC Centre for Reproductive Health, Queen’s Medical Research Institute, University of Edinburgh, Edinburgh, United Kingdom.

## Abstract

Tumor-associated macrophages (TAMs) promote metastasis and tumor cell extravasation, survival, and growth. In hepatocellular carcinoma (HCC), the presence of TAM subpopulations correlates with poor outcome. In this issue of the *JCI*, Ning et al. report on their use of cell culture, mouse models, and human data sets to investigate the interactions between aerobic glycolysis and carbonic anhydrase XII (CA12) expression in HCC. Aerobic glycolysis promoted CA12 upregulation in TAMs, which induced a protumoral phenotype to promote tumor growth and metastasis. Tumor cell factors derived from HCC samples induced CA12 upregulation in tumor-infiltrating TAMs via the HIF1α pathway. In preclinical models of HCC, CA12 inhibition reduced tumor growth and lung metastasis and reduced TAM infiltrate. Notably, dual treatment with anti-PD1 and CA12 inhibitors synergistically attenuated tumor growth and metastasis and enhanced survival compared with either treatment alone. These findings suggest that targeting CA12 in combination with immune-checkpoint blockade may provide treatment options for HCC.

## Tumor-associated macrophages have protumoral functions

Tumor-associated macrophages (TAMs) are among the most abundant immune cells in the primary and metastatic tumor microenvironments (TMEs). In most cases in human cancers, their density is correlated with poor clinical outcomes (1). Data from mouse models in a wide range of cancers have shown protumoral activities of these cells. In the primary site, TAMs enhance tumor invasion and intravasation, initiate angiogenesis, and nurture stem cells, while in the metastatic site, TAMs promote tumor cell extravasation, survival, and persistent growth (2). Importantly, in both sites, macrophages suppress immune response from cytotoxic T cells and NK cells (3, 4). Such immunosuppressive activities, if translated to humans, have the potential to impair current immunotherapies (5). In mice, TAMs are very diverse, with each protumoral function driven by identifiable subpopulations (6, 7). However, some TAMs are also likely to be antitumoral, although their activities are overwhelmed by the greater numbers of protumoral ones. Similar diversity is found in human cancers (8).

In this issue of the *JCI*, Ning et al. focus upon hepatocellular carcinoma (HCC), which has a very high frequency with unfavorable outcomes in Southern China. Structurally this cancer has a stereotypic morphology in which subpopulations of TAMs associate with pathological features of the tumor (9). The research group has previously shown that some TAM subpopulations correlate with poor outcome, while other populations associate with good prognosis (10). These data suggest that targeting and altering the protumor population phenotype could provide therapeutic options in immunotherapy.

**Figure 1:**
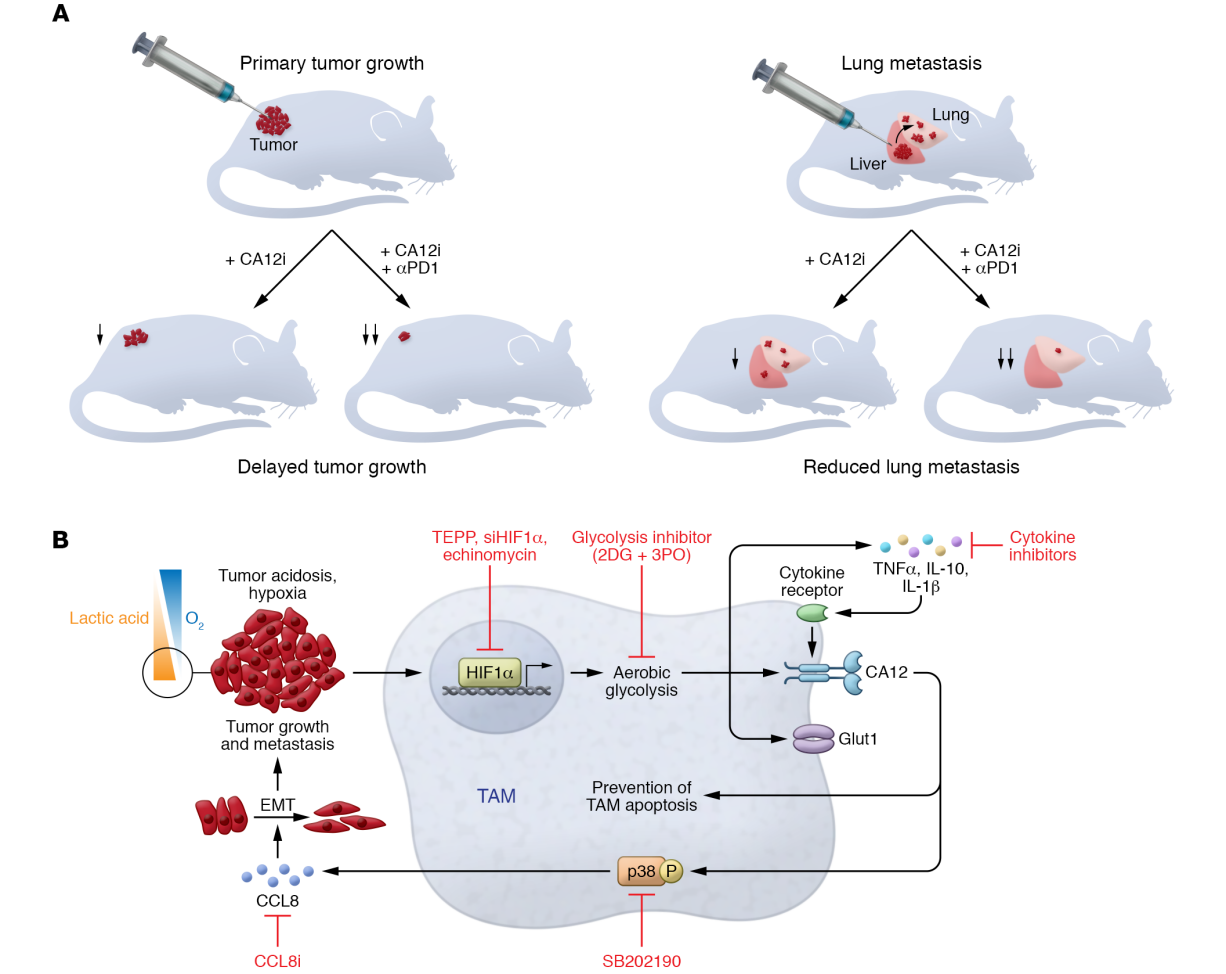
CA12 activity regulates the accumulation and functions of macrophages in TMEs. (**A**) CA12 inhibition reduces tumor growth and lung metastasis in preclinical mouse models of HCC. Targeting CA12 using CA12 inhibitors (CA12i) attenuated tumor growth and metastasis and displayed synergistic effects with anti-PD1 therapy. (**B**). Factors secreted from the tumor cells during hypoxia and acid accumulation induce aerobic glycolysis in tumor-associated monocytes/macrophages via the activation of the HIF1α-signaling pathway, indicated by increased glucose transporter GLUT1. The factors also induce CA12 and several cytokines, including TNF-α, IL-10, and IL-1β, which act in an autocrine manner to further induce CA12 expression. Notably, CA12 exhibits a protective function, promoting macrophage survival by preventing apoptosis in the acidic environment. CA12 also promotes CCL8 secretion in a p38-dependent manner, which acts on the tumor cells to induce EMT and promote tumor growth and metastasis. Targeting of this molecular pathway using relevant inhibitors could disrupt this mechanism in HCC.

Warburg first described the concept that cancer cells exhibit an alternative metabolic state to permit proliferation and compensate for the inefficiency of energy production (11). This process is a fundamental switch in metabolism that replaces cellular respiration with high rates of aerobic glycolysis, which allows high glucose uptake even in the presence of abundant oxygen, producing lactic acid instead of carbon dioxide as a by-product. The lactic acid accumulates within the TME, creating an acidic environment. Simultaneously, the TME also becomes progressively hypoxic, resulting from increased proliferation and poor vascular perfusion, and tumors develop necrotic areas. A hostile milieu results, particularly for immune cells in TME. Ning et al. identified an autocrine molecular pathway in HCC that involves carbonic anhydrase XII (CA12). CA12, induced in macrophages during aerobic glycolysis, promoted TAM survival within the acidic environment. CA12 also induced a protumoral phenotype to promote tumor growth and metastasis (Figure 1).

## Induction of CA12 in HCC-associated macrophages

Glycolysis-induced CA12 is the pivotal mediator between the acidic TME and downstream protective and tumor-promoting effects. CA12 belongs to a family of 15 carbonic anhydrases that are transmembrane enzymes involved in mediating extracellular pH. CA12 was first identified as being overexpressed in renal carcinoma cells (12, 13) and has subsequently been implicated in tumorigenesis of multiple cancers. Together, hypoxia, the acidic environment, the induction of CA12, and a poor prognosis suggest a role of CA12 in mediating tumor progression through the maintenance of the extracellular pH (14).

A previous study from the Wu and Zheng research group demonstrated that monocytes and macrophages within the TME, particularly the peritumoral region, exhibited a metabolic switch with an upregulation of aerobic glycolysis in HCC tumors (15). Building on this work, Ning et. al. analyzed patient HCC samples and demonstrated that TAMs upregulated CA12, which positively correlated with the induction of aerobic glycolysis, indicated by upregulation of the GLUT1 transporter. CA12^+^ macrophage abundance also positively correlated with poor prognosis, suggesting CA12 has a role in disease progression.

To investigate the interactions between aerobic glycolysis and CA12 expression, Ning et al. performed in vitro culture assays on CD14^+^ monocytes that were isolated from peripheral blood of healthy donors, then treated with HCC-derived supernatants. Tumor cell–derived factors induced CA12 upregulation within the tumor-infiltrating TAMs in human HCC samples. This effect was dependent on aerobic glycolysis observed in the HCC samples, as CA12 upregulation was attenuated by the addition of glycolysis inhibitors 2DG and 3PO to the in vitro assay (Figure 1).

As the HIF1α pathway is highly associated with both hypoxia and aerobic glycolysis, Ning and colleagues explored its role in glycolysis-induced CA12 expression. Indeed, while the tumor-derived secreted factors induced HIF1α in the monocytes, the inhibition of HIF1α via *siHIF1*α and echinomycin attenuated CA12 expression, demonstrating that activation of the HIF1α pathway provides a molecular link between the acidic environment and CA12 expression. It was noted that HIF1α inhibition could only partially abrogate glycolysis-induced CA12 expression, suggesting that additional mechanisms mediate CA12 expression. The HCC cells also induced an array of cytokines from the monocytes, including TNF-α, IL-10, and IL-1β, in a glycolytic-dependent manner. These cytokines further promoted CA12 expression, which was attenuated by the addition of relevant cytokine inhibitors, and thus identify an important autocrine loop within the TAMs that, together with the glycolysis-dependent effects via HIF1α, leads to sustained CA12 expression.

## CA12 protects TAMs in the acidic environment

The upregulation of CA12 provides a protective effect and promotes the survival of macrophages within the acidic TME by preventing macrophage apoptosis. In preclinical mouse models of HCC, CA12 inhibition reduced tumor growth and lung metastasis and correlated with reduced TAM infiltrate (Figure 1). Through the utilization of in vitro assays in acidic culture conditions to mimic the in situ acidic TME, Ning et al. confirmed that tumor-induced CA12 promotes the survival of macrophages in acidic conditions, which is attenuated in the presence of the CA12 inhibitors. These protective effects were not observed after macrophage depletion using three independent depletion methods, and the tumor-promoting roles were abrogated in vivo. These data confirmed the role of the TAM-expressed CA12 in promoting tumorigenesis and metastasis. The findings also confirm that the induction of CA12 plays a role in promoting TAM survival in the acidic environment.

## Role of CA12 in promoting tumor progression

In addition to the protective effects on macrophage survival, CA12 was hypothesized to exhibit protumorigenic effects due to the positive correlation between the abundance of CA12^+^ macrophages within the HCC samples and metastatic potential of tumor patients. Ning et al. demonstrated that CCL8 expression in TAMs is regulated by the hypoxic and acidic environment via CA12, thus identifying upstream metabolic regulators of CCL8 secretion within the TME. CCL8 was also upregulated in tumor-associated monocytes in a CA12-dependent manner via sustained p38 signaling (Figure 1). The abundance of CCL8^+^ TAM infiltrate is similarly increased in human breast cancer patient samples and also correlates with poor survival (8).

Within the model of HCC, secreted CCL8 acts upon the tumor cells to induce the epithelial-to-mesenchymal transition (EMT), thus potentially facilitating metastasis. CCL8 has previously been identified as a prometastatic migration factor in several mouse models, including breast cancer (8, 16, 17), glioma (18), and melanoma (19). CCL8 also acts as a chemoattractant to monocytes that differentiate to TAMs, thus providing a positive feedback loop (8). Thus, Ning et al. and previous studies support a role for CCL8 in promoting tumor cell invasion that subsequently promotes metastasis. However, targeting of CCL8 was not investigated in the in vivo HCC model; thus, the possibility that CCL8 is the effector of the increased metastasis remains unresolved.

## Conclusion

Through their utilization of a large database of HCC clinical samples, preclinical models of HCC, and complementary in vitro assays, Ning et al. described a pathway involving the induction of CA12 expression in TAMs during hypoxia. This pathway provides a protective effect to TAMs in the acidic environment and induces CCL8 expression to promote tumor cell migration and metastasis. While similar roles of CCL8 have been previously identified, Ning et al. reveal that this hypoxia-induced pathway in TAMs involves CA12 to protect TAMs in the TME.

Since the autocrine loop within TAMs originates in tumor cells, demonstrated by the effects of tumor supernatants on the TAMs, further analysis of the molecular factors upstream of HIF1α pathway activation should also be identified. While the molecular factors that induce HIF1α and subsequently CA12 were not analyzed, previous studies implicate hyaluronan as a potential mediator of the TAM phenotype. Kuang et al. demonstrated that tumor-derived hyaluronan fragments, which induce an immunosuppressive TAM phenotype (20), were responsible for activation of the glycolytic pathways in TAMs and regulated PDL1 expression (15). Similarly, CA12 is a transmembrane receptor; therefore, future investigations should identify potential ligands.

Due to the pivotal role of CA12 in both protective and tumor-promoting effects in TAMs, Ning et al. inhibited CA12 to treat HCC by disrupting the autocrine loop within TAMs, which is induced in the acidic environment. Therapeutic targeting of CA12 not only prevented the protumorigenic effects of downstream CCL8, but also increased the vulnerability of TAMs, making them susceptible to the acidic environment. Moreover, the authors demonstrated that a combination of the anti-PD1 antibody and CA12 inhibitor had a synergistic effect in attenuating tumor growth and metastasis and enhanced survival compared with either treatment alone. Increased CD8^+^ T cells were also observed in the HCC TME after CA12 inhibitor treatment. The synergistic effect with anti-PD1 therapy suggests that CA12 may also exhibit an immunosuppressive effect and that combination therapy may further reinitiate an antitumor response within the TME. Together, these data demonstrate clinical relevance for targeting of CA12 in combination with immune-checkpoint blockade to provide therapy against HCC.

## Version 1 04/01/2022

Electronic publication
